# Stiff-Person Syndrome: A Treatment Update and New Directions

**DOI:** 10.7759/cureus.11995

**Published:** 2020-12-09

**Authors:** Juan Fernando Ortiz, Mohammad R Ghani, Álvaro Morillo Cox, Willians Tambo, Farah Bashir, Martín Wirth, Gustavo Moya

**Affiliations:** 1 Neurology, California Institute of Behavioral Neurosciences & Psychology, Fairfield, USA; 2 Medicine, Universidad San Francisco de Quito, Quito, ECU; 3 Neurology, Universidad San Francisco de Quito, Quito, ECU; 4 Internal Medicine, Liaquat University of Medical and Health Sciences, Jamshoro, PAK; 5 Medicine, Carlos Andrade Marín Hospital, Quito, ECU

**Keywords:** stiff-person syndrome, stiff-limb syndrome

## Abstract

Stiff-person syndrome (SPS) is a rare and disabling central nervous system disorder with no satisfactory treatment. Muscle rigidity, sporadic muscle spasms, and chronic muscle pain characterize SPS. SPS is strongly correlated with autoimmune diseases, and it is usual to find high titers of antibodies against acid decarboxylase (GAD65). Due to its highly disabling nature and complicated treatment, we aim to create a treatment protocol through a narrative review of currently available treatments that show efficacy. We expect to facilitate management based on treatment responses ranging from first-line medication to refractory medication. We conducted a medical subject heading (MeSH) strategy. We used the term SPS with the subheading treatment: "Stiff-Person Syndrome/Therapy" [MeSH]. An initial data gathering of 270 papers came out with the initial research. After using the inclusion criteria, we had 159 articles. We excluded 31 papers for being either systematic reviews, literature reviews, or meta-analysis. From the 128 remaining articles, we excluded another 104 papers because the extraction of the data was not possible or the study outcome did not meet our demands. There are two main treatments for SPS: GABAergic (gamma-aminobutyric acid) therapy and immunotherapy. For treatment, we suggest starting with benzodiazepines as first-line treatment. We recommend adding levetiracetam or pregabalin if symptoms persist. As second-line therapy, we recommend oral baclofen over rituximab and tacrolimus. We also suggest rituximab over tacrolimus. For patients with refractory treatment, we can use intrathecal baclofen, intravenous immunoglobulin (IVIG), or plasmapheresis. We conclude that intrathecal baclofen and IVIG are more effective than plasmapheresis in patients with refractory symptoms. Propofol may be used as a bridge - temporary therapy before initiating a permanent treatment.

## Introduction and background

Stiff-person syndrome (SPS) is a progressive central nervous system disorder with a prevalence of one to two patients per million. The disease affects women two to three times more than men [1]. SMP presents with muscle rigidity, which starts in the proximal muscles and progresses distally. Patients have recurrent falls, muscle spasms, and chronic muscle pain [1]. The stiffness is due to the coactivation of agonist and antagonist muscles. It principally occurs in abdominal and paraspinal muscles producing rigidity, lumbar hyperlordosis, and postural instability. Postural instability in these patients causes recurrent falls, as mentioned before. The disease progression can also lead to limb rigidity, which can make walking incredibly tricky and, in some cases, can hinder walking completely. Psychiatric symptoms such as depression and anxiety often accompany the disorder and can be misdiagnosed as a psychiatric illness [2]. Other neurological symptoms include horizontal and vertical supranuclear gaze palsy, nystagmus, increased reflexes, and paroxysmal dysautonomic crisis [1].

We can divide SPS into III categories: type I, associated with other autoimmune conditions and usually GAD-positive; type 2, paraneoplastic, anti-amphiphysin-positive most of the time; and type 3, seronegative SPS, which is mainly idiopathic [2]. The etiology of SPS has an apparent autoimmune root, but its pathogenesis is not completely clear. It is generally associated with antibodies against glutamic acid decarboxylase (GAD) and antibodies against gephyrin, the glycine-alpha1 receptor, or gamma-aminobutyric acid (GABA) receptor-associated protein [3]. Lack of signals dependent on GABA causes rigidity and stiffness of the truncal muscles and increases sensitivity to touch or noise. Other autoimmune disorders can be diagnosed, such as Type 1 diabetes and Hashimoto’s thyroiditis, due to its autoimmune basis. In contrast, paraneoplastic SPS is generally linked to anti-amphiphysin antibodies and is associated with malignant tumors of the lung, thymus, breast, and lymphoma [3].

Diagnosing SPS requires a high index of suspicion. Nevertheless, there are no accepted criteria at the moment. The significant features that increased the suspicion of fear are the following:

1. Stiffness in the limb and axial muscles, which results in the impairment of ambulation.

2. Presence of spasms that are precipitated by movements, noises, or emotional upset.

3. Positive response to diazepam.

4. Continuous motor unit activity on electromyography (EMG), which is suppressed with diazepam.

5. Lack of other neurological signs that point out another diagnosis [4].

Significant advances have been developed in diagnosing and understanding SPS. Nevertheless, the treatment of SPS has not been clearly established, and physicians struggle to treat this condition. Because the prognosis of SPS can be devastating, it is important to establish a correct treatment protocol [4]. We conduct a narrative review of the treatment of SPS to establish a treatment protocol (first-line medication, second-line medication, refractory medication, and so on). The effectiveness and the physiological explanation of each treatment of SPS are discussed in this article.

## Review

Methods

For gathering information for this paper, we conducted a medical subject heading (MeSH) strategy. We use the term SPS with the subheading treatment: "Stiff-Person Syndrome/Therapy" [MeSH]. An initial data gathering of 270 papers came out with the initial research. For inclusion criteria, we used the following criteria:

- Papers published in English

- Papers published in the last 20 years

- Full-text papers

- Studies conducted only on humans

For exclusion criteria, we used the following criteria:

- The following types of studies were excluded: systematic reviews, literature reviews, and meta-analysis were not used.

- Papers in which data extraction was not possible.

- Publications that did not meet our study outcomes.

Results

An overview is given in Table 1 with the number of papers after applying the inclusion and exclusion criteria in the following order.

**Table 1:** Data extraction after using the inclusion and exclusion criteria

	Number of Papers
Initial gathering of information	270
Studies published in English	213
Studies conducted on humans	212
Papers published in the last 20 years	170
Use of full-text papers	159

After using the inclusion criteria, we had 159 papers. We excluded 31 papers for being either systematic reviews, literature reviews, or meta-analysis. From the 128 remaining papers, we excluded another 104 papers because the extraction of the data was not possible, or the study outcome did not meet our demands.

Discussion

We divide the discussion into two sections regarding the treatment of SPS. In the first section, we discussed GABAergic therapy with the following drugs: benzodiazepines, levetiracetam (LEV), gabapentin, propofol, and baclofen. In the second section, we discuss immunotherapy with the following drugs: rituximab, tacrolimus, intravenous immunoglobulin (IVIG), and plasmapheresis.

GABAergic therapy

Benzodiazepines

Antibodies attack GAD enzyme, which is essential in GABA manufacturing, a neurotransmitter for muscle movement control. The attack on GAD decreases GABA [5]. Benzodiazepine (such as diazepam and clonazepam) is a psychoactive drug that enhances GABA neurotransmitter's effect at the GABA receptor in enhancing inhibitory circuits. The drug has been used as sedative, muscle relaxant, and anticonvulsant [6]. Patients with classic or partial SMS and GAD-65-positive antibody have shown improvement in stiffness and spasm symptoms with long-term benzodiazepine treatment. However, this change cannot solely be attributed to monotherapy of benzodiazepine as adjunct medications were also commonly administered. Currently, diazepam is the mainstay of therapy. Most of the patients respond well to diazepam. However, the doses required for response are usually up to 60 mg daily [7]. A concern of benzodiazepine is the potential for withdrawal from the long-term and high doses used. This has triggered the use of alternative therapy, such as N-methyl-D-aspartate receptor (NMDAR) blocker therapy tizanidine. The main concern regarding the treatment with diazepam is the sedation and somnolence. Tizanidine is an alpha 2 inhibitor that also causes glutamate release inhibition, preventing glutamatergic hyperactivity, which can lead to convulsions in SMS patients [5]. Management should also be individualized in practice based on patients’ symptoms [6].

Oral and Intrathecal Baclofen

As mentioned before, benzodiazepines are first-line treatment for a patient with SPS. Oral baclofen is a second-line therapy in patients with SPS. Patients usually required high doses of both medications. Sedation and respiratory depression are an important consideration for the treatment of both conditions [8].

We found four studies that evaluated the efficacy and role of intrathecal baclofen in SPS during our search. We discovered that baclofen effectively treats SPS because being a direct agonist of GABA-B receptors does not require endogenous GABA to produce presynaptic inhibition [9]. All the studies showed how intrathecal baclofen (ITB) improved the patients’ clinical picture. In Table 2, the key findings from the included studies for the treatment of stiff-man syndrome and baclofen are given [10].

**Table 2:** Intrathecal baclofen ITB, intrathecal baclofen; IRB, institutional review board; GAD, glutamic acid decarboxylase.

Author and year of publication	Study design	Methodology	Diagnostic criteria	Conclusion
Newton et al. (2013) [9]	Case Report	The patient received an intrathecal initial baclofen dose of 50 µg/d. Gradual titration to 150 µg/d was performed.	The patient was GAD (-) and had a history consistent with SPS.	In this case report, the patient had a continued positive therapeutic effect with improved motor function at six months follow-up.
Abbatemarco et al. (2017) [8]	Case Series	Nine patients that received ITB infusion at either 25 or 50 µgs were included. The outcome measures used were pain numeric rating scale, spasm frequency scale, lower extremity modified Ashworth scale, and timed 25-feet walk. Outcomes were assessed at six and 12 months.	SPS patients identified from the IRB-approved clinical registry of ITB therapy.	ITB is effective for medically intractable spasticity due to SPS. It helps reducing oral medication doses. Patients improve their quality of life and maintain ambulation. Most patients, but not all, have a decrease in spasm frequency.
Maramattom (2010) [10]	Case Report	The patient was treated initially with oral diazepam, baclofen, and continuous infusion of diazepam. Nevertheless, symptoms persisted.	Nerve conduction negative. Electromyography show sustain contraction. GAD (+).	The patient was prescribed ITP (50 mg). His symptoms dramatically improved. The stiffness was reduced. The doses of oral medication were reduced. Ashworth scale change from five to two.

Levetiracetam

A clinical trial of LEV of three patients with an anti-GAD65 antibody level of 145 U/mL (normal < 1.0 U/mL) was conducted. Two of the patients were on IVIG and diazepam 15 mg daily, which led to an improvement in reducing the axial rigidity. However, the frequency of hypoxemic respiratory arrest attacks remained unchanged in the first patient. The second patient was on immunotherapy with IVIG, plasmapheresis, and diazepam (orally) 15 mg daily. The patient had partially eased the stiffness that improved with walking. However, on admission to the hospital, the patient showed residual motor deficits and found it very difficult to climb the stairs. The third patient with SPS had no treatment [11].

All the patients were treated with oral LEV of 500 mg twice daily. All patients had an improvement in axial rigidity and disappearance of the paroxysmal respiratory arrests within three days after starting therapy. A similar improvement was noted in the other two patients with a marked reduction in legs' stiffness and difficulty walking. On LEV treatment, all patients improved as assessed by all outcome measures. In particular, at EMG recordings, patients relaxed their musculature better [11].

In two of the patients being treated for one week with LEV, they were switched to placebo, which led to rigidity becoming worse, and paroxysms of respiratory arrest reappeared. In the third patient, the placebo was tried twice daily for 12 hours for one week before treating the patient with LEV, which led to no significant benefit.

LEV was well-tolerated. Patient with SPS could improve their muscle stiffness and paroxysmal respiratory spasms with LEV. Nevertheless, long-term therapy has not been documented. The mechanism involves the effect of LEV on SPS, which is mainly unknown. It is proposed that LEV stabilizes and strengthens GABA-A action. Also, LEV decreases the hyperexcitability in spinal cord neurons [11].

Pregabalin

In a case report, pregabalin improved the symptoms of a 71-year-old woman with marked stiffness of trunk and lower limb muscles with sudden, painful spasms for eight months. She was taking diazepam, but it was withdrawn because of excessive sedation. She had intense back pain. To relieve lumbar pain, she was prescribed pregabalin (75 mg, twice a day). The day after, rigidity and painful spasms dramatically improved, and she could walk without assistance. At three months follow-up, she still reported the improvement on rigidity and spasms; gait and osteotendinous reflexes were normal, and there were bilateral flexor plantar responses [12].

The clinical benefit was paralleled by a significant decrease in continuous motor unit activity at the EMG. Transcranial magnetic stimulation (TMS) studies in SPS demonstrated decreased intracortical inhibition consistent with GABA-A and GABA-B impaired transmission and increased intracortical facilitation, compatible with an enhancement of glutamatergic transmission [12].

The proposed mechanism of the action of pregabalin is inhibition of calcium influx and subsequent release of excitatory neurotransmitters, including glutamate and norepinephrine. As a possible mechanism of action in SPS, pregabalin might have compensated for the imbalance between inhibitory and excitatory intracortical circuits. The prolongation of the GABA-B supports this hypothesis-mediated cortical silent period (CSP) after pregabalin introduction. Interestingly, similar to osteotendinous reflexes, plantar responses were normalized after three months of treatment. One possible explanation of inverted plantar responses is that pregabalin might have reduced pathological enhancement of exteroceptive reflexes by supraspinal modulation of GABAergic transmission [12].

Propofol

In a case report, a patient with severe symptoms was taking high-dose benzodiazepines, baclofen, corticosteroids, LEV, IVIG, and IV ethanol. Nevertheless, the symptoms persist. After that, the patient received a small dose of propofol, which surprisingly improves the patient's symptoms. We also documented a patient with stiff-limb syndrome (a variant of SPS) with refractory therapy who responded well to propofol [13].

The mechanism by which propofol interacts in the nervous system is not entirely understood. Nevertheless, it is suggested that it might be related to increased GABAergic activity in the brain [13]. As it is unrealistic to have a patient with a long-term therapy to propofol, the authors suggest that propofol could be used as a bridge therapy to a more permanent intervention in patients with SPS [14].

Immunotherapy

Rituximab

It is known that more than 80% of SPS patients have elevated titer antibodies against glutamic acid decarboxylase (GAD-65), and up to 15% have antibodies to glycine receptor α-subunit. The causative link between SPS and these antibodies is uncertain, but they may be involved in the disease's pathophysiology [15].

As the B cells are the producers of antibodies, it is reasonable to use drugs that affect their function. Rituximab is a genetically engineered monoclonal antibody directed against CD20, which causes a sustained depletion of B cells and has demonstrated promising results in several autoimmune neurological diseases [15]. Accordingly, we found four articles that used rituximab in patients with SPS. Two of them were case reports: one was a letter to the editors, while the other was a double-blind, placebo-controlled trial. In Table 3, we summarized the characteristics of the studies mentioned [15-18].

**Table 3:** Studies describing the use of rituximab in SPS SPS, Stiff-person syndrome; GAD, glutamic acid decarboxylase; CSF, cerebrospinal fluid; tds: three times daily; bd: twice daily.

Author and publication year	Study design	Methodology	Conclusion
Dalakas et al. (2017) [15]	Placebo-controlled randomized trial	A total of 24 patients with confirmed SPS fulfilling established diagnostic criteria were enrolled. They were randomized to receive two bi-weekly infusions of 1 gram of rituximab or placebo (normal saline solution) while being allowed to continue receiving non-immunosuppressive drugs. The primary outcome was a change in stiffness scores at six months, and secondary outcomes were changes in heightened sensitivity and quality of life scores.	This controlled trial, defined as the largest conducted in SPS patients, showed no statistically significant difference in the efficacy measures between rituximab and placebo.
Qureshi and Hennessy (2012) [16]	Letter to the editors	A 56-year-old male with a history of type 1 diabetes, hyperthyroidism, hypertension, SPS, and progressive stiffness and painful spasms on his limbs and axial region. He took levetiracetam 500 mg bd, gabapentin 1,200 mg tds, and diazepam 10 mg tds as a symptomatic treatment for SPS. Weeks before admission to the hospital, he presented worsening of axial muscular spasms, anxiety attacks, generalized weakness, anorexia, and weight loss. On the day of admission, he had a Glasgow Coma Scale score of 6/15 and was intubated because of respiratory failure. He also received propofol and lorazepam. CSF demonstrated the presence of oligoclonal bands. Anti-GAD antibodies were positive with high titers > 1,000 U/ml. After extubation failed several trials, a tracheostomy tube was inserted for long-term ventilation. Physicians tried with plasmapheresis and oral steroids without improvement. Finally, rituximab was introduced, and his physical state improved rapidly with a decrease in stiffness.	Rituximab improved the clinical condition, but anti-GAD was reduced considerably after one year of treatment (400 U/ml). Previous reports suggested a beneficial response to immunosuppressive therapy in patients with severe disease unresponsive to benzodiazepines and/or baclofen. Lymphocyte B depletion may be beneficial in refractory forms of the disease with intensive antibody production. In this case, also, respiratory failure responded well to rituximab.
Lobo et al. (2010) [17]	Case report	A 41-year-old female presented with a history of seven years of progressive rigidity to the abdominal wall and paravertebral muscles, with painful spasms in the lower back region, increased tonus on shoulders, and exaggerated lumbar lordosis. Physicians investigated SPS as a possible diagnosis and tested for anti-GAD, which was positive with high titers. Electromyography also supported the diagnosis. The patient was treated with intravenous diazepam and botulinum toxin type A. Trials to change her IV medication to per os was not well-tolerated. After an extensive discussion, specialists decided to try rituximab for the treatment of SPS.	Two days after the rituximab’s infusion, her muscular tonus decreased, prompting reduced intravenous benzodiazepine doses. After the second dose, 15 years later, she tolerated oral diazepam without spasms. This patient presented clinical improvement, but unlike other reports, anti-GAD titers continue rising despite rituximab. The authors suggested that the anti-GAD antibody titer may have no relation to the disease’s clinical presentation.
Baker et al. (2005) [18]	Case report	A 41-year-old with a diagnosis of SPS admitted to the hospital as an emergency with prolonged and painful extensor spasms affecting the neck, back, arms, and legs. She had been bed-bound for several months. Regular medication included baclofen 40 mg tds, dantrolene sodium tds, fentanyl 25 mg path twice weekly, and up to 80 mg of parenteral diazepam per day, up to 25 mg of diamorphine. Anti-GAD antibodies were positive. Antispasmolytics and various disease-modifying treatments were tried over the year without remarkable benefit. During this hospitalization, she received rituximab.	Fifteen days after the infusion of rituximab, stiffness began to resolve, and the patient was able to shower herself for the first time in more than two years. Anti-GAD titers became undetectable 17 days after treatment. After the sixth week, she was re-admitted and treated with another course of rituximab and mycophenolate mofetil. Her condition improved and was able to stand and walk with help, sit, and shower. This report is considered the first of the successful use of rituximab in the treatment of SPS.

There is a lack of evidence to establish if rituximab improves patients' symptoms and outcomes with SPS. Although the case reports and letters to the editors showed benefit in the patients described, the placebo-controlled randomized trial, a most complex study, demonstrated no statistically significant difference. It is important to consider that even if the series represents the largest study in SPS in the clinical trial, the number of patients involved may still be small. More investigation appears to be crucial to determine whether the anti-B cell agents are useful or not.

Tacrolimus

Two patients with unsuccessful treatment of other modalities were treated with tacrolimus. Tacrolimus belongs to the same class of medication as cyclosporine (calcineurin inhibitor) but is more potent. Tacrolimus inhibits the activity of T cells [19]. At the start of the treatment, both patients had muscle stiffness, spasm, and high GAD antibodies' titters. The first patient was treated with tacrolimus and intravenous Ig. The second patient was treated with prednisone and tacrolimus. The patients’ symptoms improved after four weeks, and the antibody titters decreased. The mechanism by which tacrolimus interacts is very interesting. Tacrolimus decreases the levels of IL-2, which impaired the function of T helper cells. If the T helper cells can conduct their function, there will be less activation of B cells to produce antibodies.

Interestingly, tacrolimus also seems to have a neuroprotective effect [19]. At the moment, there is more evidence of efficacy with rituximab over tacrolimus. We can prefer at the moment rituximab over tacrolimus. However, new studies could change our perspective in the future.

Intravenous Immunoglobulin Therapy

The mainstay treatment of SPS is the control of symptoms. Patients with severe or unsatisfactory symptoms must initiate immunomodulators like IVIG other than conventional therapies [20]. A double-blind placebo-controlled study used 2 g/kg of IVIG twice daily for three months vs. half-normal saline as a placebo. Three months after the end of the course, the study assessed the patients based on the number of stiff areas. Of 16 patients in the study, six received IVIG and demonstrated a significant improvement in the tested clinical parameter. Patients could walk without assistance, stopped falling, crossed the street, and no spasms during household chore activities were noted.

Furthermore, those assigned to the placebo group did not show significant changes; however, after receiving IVIG, these patients improved significantly but worsened after the placebo. In terms of safety, IVIG was safe and effective, as clinical outcomes showed [20]. Likewise, one cross-over study used IVIG for three months vs. placebo. The study was followed by a one-month washout period and then three months of therapy with an alternative regimen. Patients under the IVIG regimen had lower stiffness scores over the placebo group. IVIG regimen was safe and had more efficacy over the placebo group as well. The improvement duration of IVIG therapy was six weeks to one year.

Moreover, there was a drop of IgG titers (anti-GAD), as was not the case in the placebo group. The anti-GAD levels did not correlate with the severity of the disease [7]. Another small clinical trial of six patients found that IVIG improves the patients' quality of life. This study showed improvement in pain subscores, social functioning, mental health, and energy level [21].

Plasma Exchange (Plasmapheresis) Therapy

SPS presents with a myriad of outcomes; some patients respond to initial treatment, while others fail to respond. After the use of several treatments and the patients continue to fail to improve, plasma exchange therapy could be another option. Nevertheless, conflicting results have emerged from therapy from plasmapheresis [22]. Plasmapheresis is usually conducted in one cycle with five sessions of plasma exchange. A study conducted by Casa-Fages et al. found two patients who responded to therapy and had an insufficient response to other therapies before. Despite improvements in both patients' symptoms, the anti-GAD levels remain elevated after treatment [22].

It is suggested that plasmapheresis' therapeutic effect is related to the elimination component of the immune system. Among them are the complements, cytokines, or other modulatory components of the immune system [22]. Another case series demonstrated the short-term effectiveness of plasmapheresis in patients with refractory treatment to IVIG [23]. In a clinical trial with 10 patients with SPS, six patients continue the treatment chronically on an outpatient basis. However, three patients have complete remission of their symptoms, while seven patients only partially relieve symptoms [24]. Studies suggest that plasma exchange may be useful as adjuvant therapy, mainly in patients with no IVIG regimen response. In an acute and long-term setting such as respiratory depression, plasmapheresis could save the patient's life, although with variable response [24]. 

Establishing a treatment protocol

We suggest starting with benzodiazepines because part of the SPS criteria is the improvement of symptoms with benzodiazepine and is also the oldest treatment. So, benzodiazepines are both useful for therapy and diagnosis [7].

We suggest adding or switching LEV and pregabalin as first-line treatment. Both drugs have low toxicity levels and proven effectiveness in patients with SPS [11,12]. As the second line, we suggest oral baclofen if the symptoms do not improve. We recommend trying immunotherapy or intrathecal baclofen if refractory symptoms persist [9,10]. Propofol should be used as a bridging treatment when the patient has refractory symptoms and anticipates switching to a more permanent therapy, thus using propofol as the short-term treatment of the symptoms [13]. Regarding immunotherapy, we discussed four available options: rituximab, tacrolimus, plasma exchange therapy, and IVIG.

Benzodiazepines remain the preferred first-line treatment. However, immunotherapy can be used as a second-line therapy due to the side effects. We could start with rituximab because there are fewer studies on tacrolimus [15-19]. Suppose one or both treatments fail; in that case, we suggest to start with IVIG over plasmapheresis [21]. Plasmapheresis, in our findings, had mixed results compared with IVIG, which had consistent effects and improved symptoms of SPS [24].

## Conclusions

As described, medical treatment for SPS has two main groups: GABAergic therapy and immunotherapy. GABAergic therapy includes benzodiazepines, pregabalin, levetiracetam, and baclofen. In comparison, immunotherapy includes rituximab, tacrolimus, plasma exchange, and IVIG. The mechanism of propofol is not very clear. Benzodiazepines and pregabalin enhance GABA transmission by acting as direct agonists of GABA-A receptors. In comparison, baclofen plays a role in the GABA-B receptor. Levetiracetam is not only a calcium channel blocker, it also has GABAergic activity. Rituximab causes a sustained depletion of B cells and tacrolimus impairment of T helper cell function. Both of these mechanisms are directed to inhibit anti-GAD antibody production. Intravenous immunoglobulin has an exciting mechanism with immunomodulatory effects based on antigen-antibody interactions. Finally, plasmapheresis causes depletion of circulating antibodies or immunocomplexes and pro-inflammatory proteins.

In conclusion, benzodiazepines should be the initial treatment in persons with SPS. We recommend adding levetiracetam or pregabalin if symptoms persist. As second-line therapy, we recommend oral baclofen over rituximab and tacrolimus. Baclofen has fewer side effects than rituximab and tacrolimus. Between rituximab and tacrolimus, we recommend rituximab because it has higher efficacy. For patients with refractory treatment, we can use intrathecal baclofen, IVIG, or plasmapheresis. Intrathecal baclofen and IVIG are more effective than plasmapheresis in patients with refractory symptoms. Finally, we found that propofol is effective in patients with refractory symptoms; nevertheless, it is not practical, and we can only use it for the short term. Before using another long-term therapy, we suggest using propofol as a bridge before using more permanent treatment.
